# Patterns of mixed *Plasmodium* species infections among children six years and under in selected malaria hyper-endemic communities of Zambia: population-based survey observations

**DOI:** 10.1186/s12879-015-0935-7

**Published:** 2015-05-02

**Authors:** Lungowe Sitali, James Chipeta, John M Miller, Hawela B Moonga, Nirbhay Kumar, William J Moss, Charles Michelo

**Affiliations:** Department of Biomedical Science, University of Zambia, School of Medicine, Lusaka, Zambia; Department of Paediatrics and Child Health, School of Medicine, Malaria Research Unit (SMUTH-MRU), Lusaka, Zambia; Department of Paediatrics and Child Health, University of Zambia, School of Medicine, Lusaka, Zambia; PATH-Malaria Control and Elimination Partnership in Africa (MACEPA), National Malaria Control Centre, Lusaka, Zambia; Ministry of Health, National Malaria Control Centre, Lusaka, Zambia; Department of Tropical Medicine, Tulane University School of Public Health and Tropical Medicine, New Orleans, USA; John Hopkins Malaria Research Institute, Bloomberg School of Public Health, Johns Hopkins University, Baltimore, MD USA; Department of Public Health, University of Zambia, School of Medicine, Lusaka, Zambia

**Keywords:** Malaria, Mixed infections, Non-falciparum infections, Prevalence, Zambia

## Abstract

**Background:**

Although malaria is preventable and treatable, it still claims 660,000 lives every year globally with children under five years of age having the highest burden. In Zambia, malaria rapid diagnostic tests (RDTs) that only detect *Plasmodium falciparum* are the main confirmatory means for malaria diagnosis in most health facilities without microscopy services. As a consequence of this *P. falciparum* species diagnostic approach, non-*falciparum* malaria is not only under-diagnosed but entirely missed, thereby making the exact disease burden unknown. We thus investigated the prevalence of various *Plasmodium* spp. and associated burden of infection in selected communities in Zambia.

**Methods:**

Data from two malaria hyper-endemic provinces (Eastern and Luapula) of the 2012 National Malaria Indicator Survey (MIS), conducted between April and May 2012, were used. The MIS is a nationally representative, two-stage cluster survey conducted to coincide with the end of the malaria transmission season. Social, behavioural and background information were collected from households as part of the survey. Thick blood smears, RDTs and dried blood spots (DBS) were collected from children below six years of age. Slides were stained using Giemsa and examined by microscopy while polymerase chain reaction (PCR) was used to analyse the DBS for malaria *Plasmodium* spp. Multivariate logistic regression was employed to examine the association between background factors and malaria.

**Results:**

Overall, 873 children younger than six years of age were surveyed. The overall prevalence of *Plasmodium* spp. by PCR was 54.3% (95% CI 51–57.6%). Of the total *Plasmodium* isolates, 88% were *P. falciparum*, 10.6% were mixed infections and 1.4% were non-*falciparum* mono infections. Among the mixed infections, the majority were a combination of *P. falciparum* and *P. malariae* (6.5% of all mixed infections). Children two years and older (2–5 years) had three-fold higher risk of mixed malaria infections (aOR 2.8 CI 1.31–5.69) than children younger than two years of age.

**Conclusion:**

The high prevalence of mixed *Plasmodium* spp. infections in this population stresses review of the current malaria RDT diagnostic approaches. The observed less incidence of mixed infections in children under two years of age compared to their older two-to-five-year-old counterparts is probably due to the protective maternal passive immunity, among other factors, in that age group.

## Background

Malaria remains a significant public health problem in many countries throughout the world, especially in sub-Saharan Africa, despite being preventable and treatable [1]. In 2010, the World Health Organisation (WHO) estimated approximately 219 million malaria cases and 660,000 deaths, with 90% of the deaths occurring in Africa [2]. Approximately 40% of the world’s population lives in areas that have some risk of malaria [3]. The distribution of *Plasmodium* spp. is not clearly known, although estimates of the global distribution of *Plasmodium falciparum* and *P. vivax*, the most predominant malaria Plasmodium spp., are widely cited [4-6]. Mueller *et al* [6] reported that the global numbers for *P. malariae* and *P. ovale* are unknown and the burdens *of P. ovale* and *P. malariae* are even more underrepresented in surveys where microscopy is employed since there are morphological resemblances between *P. ovale* and *P. vivax.*

Non-falciparum malaria may be present in Zambia given that *P. malariae* cases are routinely reported and the anecdotal prevalence is estimated to be approximately 2%. Furthermore, Blossom *et al.* [7] described a case of a 23-year-old woman who, after a trip to Zambia, presented with a prolonged illness characterized by fevers of up to 38.1°C and fatigue. Malaria smear and antibody tests were negative but polymerase chain reaction (PCR) showed the presence of *P. vivax*. Low prevalence of *P. vivax* on the African continent is attributed to high prevalence of the Duffy negative factor, a gene inherent among the majority of indigenous Africans that is otherwise responsible for refractoriness to *P. vivax* [8-10].

Infections with more than one *Plasmodium spp.* are possible and are called mixed infection. Mixed malaria species infections are often not recognized or are underestimated by microscopists [11,12]. In Asia, surveys usually report that greater than 2% of infections are mixed, whereas therapeutic studies in *P. vivax* or *P. falciparum* malaria have demonstrated a high prevalence of up to 30% of mixed infections. Other malaria species have also been reported during convalescence, suggesting covert co-infections [13]. In Manhica District of Mozambique, sharing borders with Zambia’s Eastern Province, *P. malariae* and *P. ovale* occurred almost exclusively in mixed infections [14]. This situation may be similar to the Zambian scenario as the geography and climatic factors are similar. Although this has not been documented, Zambian technologists and scientists often see and report a few cases of mixed infection routinely.

The Zambia National Malaria Control Programme (NMCP) has been at the forefront of scaling up malaria interventions over the past decade, including the early adoption of artemisinin-based combination therapy and use of rapid diagnostics tests (RDTs) for clinical management of malaria. The 2011-2015 National Malaria Strategic Plan calls for the creation of malaria free zones during its current five-year span. Therefore, understanding the extent to which current tools are able to achieve these goals is important. It follows that further understanding of the distribution of local malaria parasite species is important for developing appropriate preventive as well as diagnostic and treatment option. Unfortunately, many malaria-endemic countries, especially in sub-Saharan Africa, are just beginning to understand the relative levels of prevalent malaria parasite species as malaria control programmes begin to expand in the pursuit of malaria elimination. With the anecdotal evidence that *P. falciparum* is the predominant cause of malaria (98%), Zambia has relied on the exclusive use of histidine-rich protein 2 (HRP2) or *P. falciparum*-based antigen detection RDTs. [15].

This study sought to determine the proportion of mixed *Plasmodium* spp. causing malaria in Eastern and Luapula provinces of Zambia using stored samples from the Malaria Indicator Survey of 2012, conducted to monitor the progress of malaria control implementation efforts by the National Malaria Control Program (NMCP). This information is necessary to guide and inform malaria management and control strategies as well as provide country-specific evidence for strategic malaria diagnostic service choices and treatment options.

## Methods

### Study design

Data and samples from MIS conducted between April and May 2012 for two provinces in Zambia were utilized. MIS’s have been conducted biannually since 2006 and are used by the NMCP to evaluate progress in scaling up malaria control interventions and to monitor levels of malaria parasite prevalence among children aged five years and younger [16]. The 2012 MIS was the fourth in the series, the first being in 2006, the second in 2008 and the third in 2010. Details of MIS findings are reported elsewhere [17-19].

Study samples from children under six years of age were used from two provinces, Eastern and Luapula. These two provinces were noted to be hyper-endemic for malaria, with the highest levels of malaria parasite prevalence among sampled children during the 2012 MIS study [16]. For this study, all records with complete information on relevant socio-demographic characteristics of household members as well as verifiable records of RDT results, blood smears and dried blood spots (DBS) for all children below six years of age were included in the study. The RDTs used in this survey were HRP2 antigen-based SD Bioline (Standard Diagnostics, INC) RDTs.

### Polymerase chain reaction (PCR) protocol

Identification of *Plasmodium spp*. was performed using nested PCR, performed at the National Malaria Control Centre molecular laboratory with further validation on random samples at Tulane University. DNA was extracted using the chelex method [20] and the nested PCR assay was adapted from Nsobya *et al* [21].

The protocol was designed such that in the first reaction a conserved region for the four species was amplified (*P. falciparum, P. vivax, P. ovale and P. malariae*). In the second reaction, different primers were run for the four species separately. The band sizes of the products were as follows: *P. falciparum* 205 bp, *P. malariae* 140 bp, *P. ovale* 800 bp and *P. vivax* 120 bp.

The PCR mixture for the first reaction contained 12.5 μL of a 2X master mix containing Taq buffer (10 mM Tris–HCl, pH 8.3, 50 mM KCL, 1.5 mM MgCl_2_), 200 μM of each dNTP 2.5 units of Taq DNA polymerase, PCR primers (10 μM of rPLUf and rPLUr) and 5 μL of DNA template and water in a final volume of 25 μL. In the second reaction, the same master mix was used but with different primer concentration (10 μM of each of the species-specific primers in separate tubes), 2 μL DNA template from the first reaction in a 25 μL reaction volume. The PCR was run in a gene Amp PCR system 9700 (Applied Biosystems thermocycler, Foster City CA, USA) under the following conditions: the first reaction had denaturation at 94°C for one minute followed by 35 cycles at 94°C for one minute, 58°C for two minutes and 72°C for five minutes; the second reaction (with four tubes for each primer) had denaturation at 94°C for one minute, followed by 30 cycles at 94°C for one minute, 58°C for two minutes and 72°C for five minutes. The final cycle was followed by an extension time of five minutes at 72°C. A 2% agarose gel stained with ethidium bromide was run for visualisation of the PCR product [20].

### Data and statistical analysis

Demographic and laboratory data of the participants’ records was analysed with Stata version 11 (College Station, Texas, USA) with the cluster effect accounted for in the analyses. The Mantel-Haenszel chi square test (*x*^2^), and in some cases Fisher’s exact tests for proportions, were used to test for independence. Multivariate logistic regression was used to examine the association between background factors with malaria between comparable categories. The distribution of age as a continuous variable conformed to normality as assessed by probability plots.

### Ethical approval

The 2012 MIS survey protocol received clearance by the University of Zambia Biomedical Research Ethics Committee as a continuation of the 2006, 2008 and 2010 surveys. The reference for the Malaria Indicator Survey it was 002-03-12. In addition, participation in the MIS questionnaires and malaria testing were based on informed consent from their respective parents or guardians as all children in the survey were below six years of age. Respondents were counselled and informed that the testing was purely for research purposes and was to be handled anonymously. However, respondents were informed of their test results and available treatment was provided based on national malaria treatment standard care guidelines. For this study of mixed infections, additional approval was sought from the committee; reference number was 001-10-12.

## Results

### Socio-demographic characteristics

The *de facto* eligible population comprised only children younger than six years who had complete records and were tested for malaria infection. Thus, only 873 children were included in the final analysis. Of these children, 47% were male and 53% were female. There were 504 (57.7%) children from Eastern Province and 369 (42.3%) from Luapula Province. Larger proportions (95.7%) of these children were classified according to the national census as living in rural clusters, with only 4.4% living in an urban cluster (Table 1).

**Table 1:** **Socio-demographic factors for the study participants in the Luapula and Northern Provinces in Zambia**

**Total number of children test =873**		
**Characteristics**	**N**	**% (CI)**
**Gender**		
Male	410	47.0 (43.6–50.2)
Female	463	53.0 (49.7–56.4)
**Age of children**		
<1 year	132	15.1 (12.7–17.5)
1 yr	144	16.5(14.0–19.0)
2 yrs	151	17.3(14.8–19.8)
3 yrs	178	20.4(17.7–23.1)
4 yrs	156	17.9 (15.3–20.4)
5 yrs	112	12.8(10.6–15.1)
**Province**		
Eastern	504	57.7 (54.4–61.0)
Luapula	369	42.3 (39.0–45.5)
**Residence**		
Urban	38	4.4 (3.0–5.7)
Rural	835	95.7 (94.3–97.0)
**Age of Mother**	47	6.0 (4.3–7.7)
15–19	207	26.4 (23.3–29.5)
20–24	203	25.9 (23.0–28.9)
25–29	254	32.4 (29.1–35.6)
30–39	74	9.4 (7.4–11.5)
**School level of mother**		
Low-Below grade 5	420	53.5 (50.0–57.0)
High- Grade 5 and above	365	46.5 (43.0–50.0)

### Prevalence

**Figure 1:**
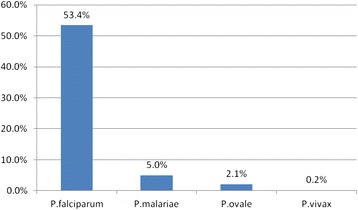
Prevalence of *Plasmodium* species infections in Eastern and Luapula provinces in Zambia.

Overall, the prevalence of malaria infection using PCR was 54.3% (95% CI 51–57.6%) (Figure 1), with Luapula Province accounting for a higher burden than Eastern Province (55.6% vs. 52.6%, respectively). From the total positive samples, the predominant species was *P. falciparum* (88.3%) with 11.6% due to non-*falciparum* species and 10.3% were mixed infections (Table 2). The observed mixed infections were in five categories or combinations. Most children were found to have a combination of *P .falciparum* and *P. malariae* (6.5%); *P. falciparum*, *P. malariae* and *P. ovale* (6%); and *P. falciparum* and *P. ovale* (2.1%). The combinations of *P. falciparum*, *P. malariae* and *P. vivax* (0.2%), and *P. falciparum* and *P. vi*vax (0.2%) were rare (Table 2).

**Table 2:** **Differential overall prevalence of mixed and mono infections in Eastern and Luapula Provinces combined**

**Infection pattern**	**No.**	**Percentage**
**P.f**	**419**	**88**
**P.m**	**4**	**1**
**P.o**	**2**	**0.4**
**Pf + Pm + Po**	**6**	**1.3**
**Pf + Pm + Pv**	**1**	**0.2**
**Pf + Pm**	**31**	**6.5**
**Pf + Po**	**10**	**2.1**
**Pf + Pv**	**1**	**0.2**
**Total**	**474**	**100**
**Total combinations**	**55**	10.3*

Note: All positives is the n = 474, *10.3%, is the percentage for all mixed infection combination.

Key: **Pf**-*Plasmodium falciparum*; **Pm**- *Plasmodium malariae*; Po*- Plasmodium ovale*; **Pv**- *Plasmodium vivax.*

### Factors associated with mixed plasmodium infections

Assessment of factors associated with mixed *Plasmodium* infections (Table 3) showed an association between mixed infections with province and age of the child. Children older than two years of age were almost three times more likely to have mixed malaria infections than children younger than two years of age (aOR 2.8, 95% CI, 1.31–5.69). This association was stronger in Luapula province where likelihood of children having mixed infection was four-fold higher (aOR 4.2, 95% CI 2.00–8.73) than in children residing in Eastern Province.

**Table 3:** **Results logistic regression model for malaria mixed infection for the study participants from Luapula and Northern Provinces in Zambia**

**Malaria mixed parasites infection**	**n = 873**		
**Characteristic**	**Prevalence**	**aOR**	**95% CI**
**Age in years**			
0 > 2	2.8%	1	
2–5	8.3%	2.80	1.31–5.69
**Gender**			
Male	46.4%	1	
Female	53.6%	0.9	0.50–1.88
**Age of the mother in years**			
15–24	3.5 %	1	
25–39	5.3 %	1.4	0.61–3.13
40–49	8.1 %	2.6	0.83–7.84
**Province**			
Eastern	3.0 %	1	
Luapula	9.2 %	4.2	2.00–8.73
**Ownership of Mosquito net**			
No	58.0%	1	
Yes	53.4%	0.5	0.14–1.79

## Discussion

The study showed a significantly high prevalence of malaria in two provinces of Zambia, demonstrating for the first time to our knowledge the prevalence of the four major *Plasmodium* species in the studied population. Although the malaria prevalence is predominantly due to *P. falciparum*, the prevalence of non-*falciparum* mixed infections was significantly higher than previously reported. This high prevalence partly explains why malaria still remains one of the leading causes of morbidity and mortality in Zambia despite the current control and management measures. The high prevalence of malaria due to all four species and in both mono and mixed infections patterns clearly reveals not just the presence of non-*falciparum* species but also the dynamic and evolving nature of the malaria epidemic. The 11.6% is actually close to the anecdotal level of non-*falciparum*-only infections known, but mixed *falciparum* infections account for a more significant contribution of the total infection prevalence than previously considered. These findings suggest that there is need for continued monitoring of non-*falciparum* infection prevalence in this population so as to decide when species-specific RDTs should be introduced for diagnostic purposes. The species-specific diagnosis will be important when the prevalence of malaria in this population reduces and as the country moves towards malaria elimination. In addition, Coartem, the first-line treatment drug in Zambia, does not clear Plasmodium hypnozoites and radical treatment with primaquine, effective against hypnozoites may play an important role in the control and eventually elimination of *vivax* and *ovale* malaria, and malaria consequently [22].

Differential species burden patterns observed were not surprising as similar observations have been reported elsewhere [14,23]. However, the fact that non-*falciparum* malaria cases were present in the study population necessitates the need to have diagnostic tools or approaches at the national level that should facilitate the detection of such non-*falciparum* malaria cases. While it should not necessarily be the primary diagnostic tool, diagnostic approaches that have the capacity to further investigate and detect non-*falciparum* malaria may become important in future. For health facilities where microscopy services are present, there is a need to conduct refresher courses for laboratory staff and microscopists with an emphasis on *Plasmodium* species-wide microscopy so as to strengthen non-*falciparum* malaria diagnosis as has been reported and advocated elsewhere [7,10,24].

Among the factors associated with mixed malaria infections, age was found to be a predictor. This is in agreement with a similar study by Guerra-Neira et al [25] who reported an inverse correlation between of age and multiplicity of Plasmodium infection, with children under five years of age having higher frequency of mixed *Plasmodium* malaria compared to individuals older than five years of age. Children younger than two years in our study were found to be less likely to have mixed malaria infections compared to those aged from two to five years. This could be partly explained by the maternally derived antibodies believed to offer protection from infections. Riley et al reported that neonates and infants are relatively protected from clinical malaria, although the mechanism of the protection is not clearly understood [26]. Indeed, the dynamics of immunity in neonates, infants and toddlers is quite remarkable resulting in variable susceptibility to infection [27,28]. Noteworthy here is that, unlike the study by Guerra-Neira et al [25], our study population was limited to children younger than five years of age. In addition, these findings could be attributed to the use of insecticide-treated nets (ITNs) in that younger children tend to sleep under ITNs as breast-feeding infants but, at the age of two-to-three years, they are weaned off from breasting feeding and may not sleep under an ITN.

## Conclusion

This study reveals the presence of mixed infections in two provinces of Zambia with an overall prevalence of 10.3%. The major factor associated with mixed infection was the age of the child. This might call for repackaging of control and management measures at national level. The presence of non-*falciparum* infections might vary with transmission patterns, demographic trends and geographical contrasts. There is the need for these to be monitored through a functional surveillance system so as to understand the epidemiological profile. Given that most of the non-*falciparum* malaria cases occur as mixed infections, the use of HRP2-based RDTs can still continue in health facilities but need to be strengthened with improved and enhanced microscopy to facilitate detection of the non-*falciparum* malaria cases. Finally, there is a need for further studies to have a complete, detailed profile and epidemiology of non-*falciparum* species of malaria in the country, especially in the era of malaria elimination activities in the country and region.

## Abbreviations

HRP2: Histidine rich protein 2
DBS: Dried Blood Spot
DNA: Deoxyribonucleic Acid
ITNs: Insecticide-treated nets
NMCC: National Malaria Control Centre
NMCP: National Malaria Control Programme
MIS: Malaria Indicator Survey
PCR: Polymerase chain reaction
RDTs: Rapid diagnostic tests
WHO: World Health Organisation
